# Complete androgen insensitivity syndrome or testicular feminization: review of literature based on a case report

**DOI:** 10.11604/pamj.2016.25.199.10758

**Published:** 2016-11-28

**Authors:** Regragui Souhail, Slaoui Amine, Abounouh Nadia, Karmouni Tarik, El Khader Khalid, Koutani Abdellatif, Ibn Attya Ahmed

**Affiliations:** 1 Department of Urology B, Ibn Sina University Hospital, Rabat, Morocco; 2 Department of Gynecology Obstetrics, Maternity ibn Sina, Rabat, Morocco

**Keywords:** Testicular, feminization, androgen, insensitivity, laparoscopy

## Abstract

Testicular feminization, or the androgen insensitivity syndrome, is a rare disease. Because of various abnormalities of the X chromosome, a male, genetically XY, has some physical characteristics of a woman or a full female phenotype. Indeed the androgen insensitivity syndrome occurs because of a resistance to the actions of the androgen hormones, which in turn switches the development towards the aspect of a woman. We report a case of complete androgen insensitivity syndrome in a 30 years old woman who presented primary amenorrhea. We aim to improve our knowledge of this illness from the data that provides us this study, and a review of the literature.

## Introduction

Testicular feminization or androgen insensitivity syndrome is a rare disease. Because of various abnormalities of the X chromosome, a male, genetically XY, has some physical characteristics of a woman or a full female phenotype. We report a case of complete Androgen insensitivity syndrome in a 30 years old woman with primary amenorrhea. From this observation, we present the clinical and pathological aspects and then report our therapeutic strategy.

## Patient and observation

**Figure 1:**
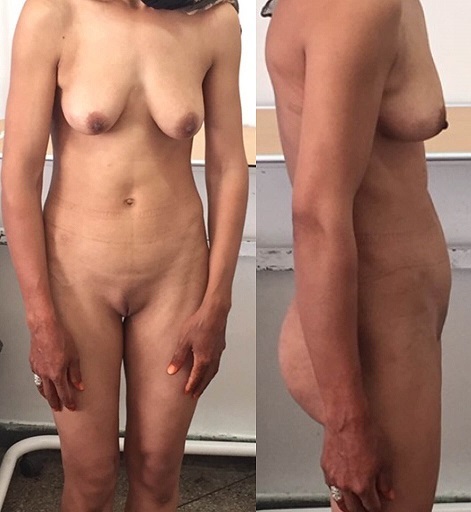
Front and side view of the patient

**Figure 2:**
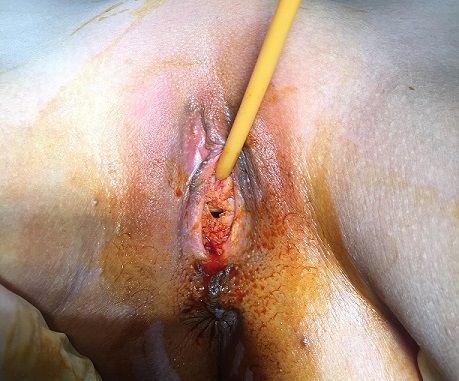
Clinical aspect of the vagina

**Figure 3:**
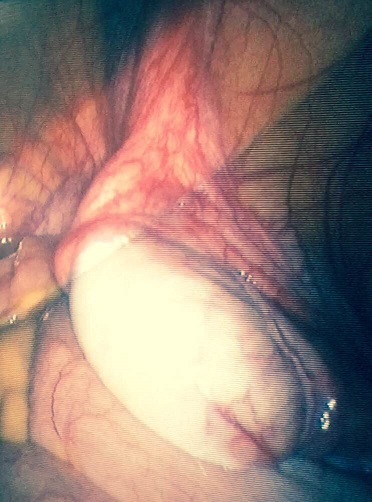
Intra- abdominal testes: laparoscopic aspect

**Figure 4:**
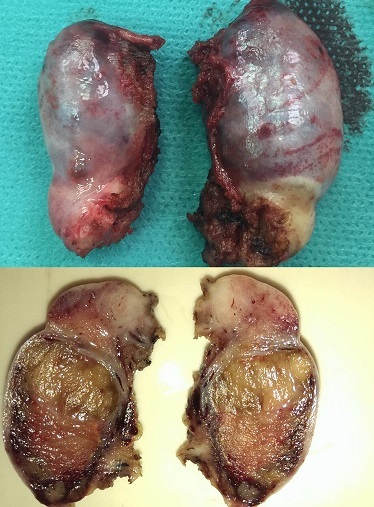
The excised testis: Macroscopic aspect

**Figure 5:**
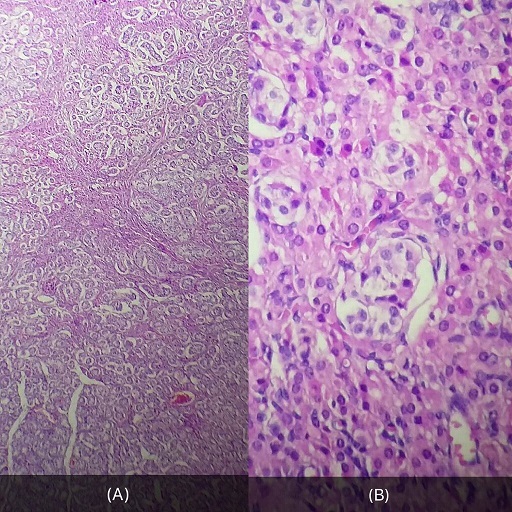
Testes: atrophy of the seminiferous tubules; Histopathological aspect, HE staining: (a) ob: 10×; (b) ob: 40×

We report a case of a 30 year old woman who was admitted to a gynecology department for primary amenorrhea. Clinical examination revealed a female phenotype: the breasts were normally developed, however, the labia was small and we notice the absence of axial hair, pubic and groin. Gynecological examination puts in evidence the hymen, a short vagina (2cm) and no uterus (Figure 1, Figure 2). Sexual hormones in blood were measured: Gonadotropins were found normal (FSH 6.64 mUI/mL, LH 26.65 mUI/mL), so the progesterone (6.89 nmol/L) and estradiol (58 pmol/L), nevertheless the testosterone 2G was high for a woman (58.61 ng/mL). The karyotype was mapped and highlighted a male genotype 46XY. Because of the lack of androgen receptor, gene abnormality research and an androgen binding test in a genital skin fibroblast were not realized. Thus the pelvic MRI revealed the absence of uterus and ovaries, hypoplastic vagina, and intra-abdominal testes. Later on, she was referred to our urology department to undergo a laparoscopic removal of the undescended testes in order to avoid risk of malignancy. The testes were found inside the abdominal cavity. They were subsequently dissected and removed (Figure 3, Figure 4). Histopathology revealed two testes with atrophic seminiferous tubules containing only Sertoli cells, associated to a Leydig cells hyperplasia. Hopefully, no signs of testicular cancer were identified (Figure 5). Postoperative evolution was uneventful and the patient left hospital after one day. Mentally, the patient kept seeing herself as a woman. So the decision to reveal the truth was difficult. Therefore, we opted for the establishment of psychological support and refer her to the endocrinology department to benefit from estrogen substitution therapy.

## Discussion

During the 6th week of the male fetal development, the testes begin their differentiation. These phenomena are under the influence of the SRY gene located on the Y chromosome. Leydig cells appear through the end of the 8th week and they start producing testosterone. Afterwards, under the influence of androgen hormones, the rest of the male sexual characteristics slowly take place (including the testicular translocation to the scrotum) [1]. The Anti-Müllerian hormone secreted by Sertoli cells prevents the development of the Müllerian ducts into the uterus and other Müllerian structures. If no hormone is produced from the gonads, the Müllerian ducts automatically develop, while the Wolffian ducts, which are responsible for male reproductive parts, automatically die. Testosterone affects cells through androgen specific nuclear receptors. The proteins are encoded by a gene located on the proximal long arm of the X chromosome, specifically locus Xq11-Xq12 [1]. However, not all mutations result in a defective androgen receptor. Indeed, this protein comprises of several functional parts: the transactivation domain, the DNA- binding domain, the hinge, and the steroid-binding domain. In addition, the transactivation domain, which is the most likely affected, represents more than half of the receptor. This translates to an inability of all cells to recognize and use testosterone, thus leading to androgen insensitivity syndrome [2]. But, there are some cases of testicular feminization that occur without a mutated androgen receptor gene. Other much less frequent causes are: mutant steroidogenic factor-1 protein; a deficit in the transmission of a transactivating signal from the N-terminal region of the normal androgen receptor to the basal transcription machinery of the cell [3]. The main concern with complete androgen insensitivity syndrome is the diagnosis. Indeed, it requires many tests, some quite rare and frequently unavailable, so therefore it is often uncertain [4]. Complete Androgen Insensitivity Syndrome, with its female phenotype, is overlooked at birth, and is diagnosed at puberty, when the patient reports primary amenorrhea such as in our case. Nevertheless, it could be found even prenatally if the karyotype is determined from the amniotic fluid, and the genetic sex would be verified through ultrasound. However, this is rarely the case. After discovering primary amenorrhea, blood levels of different sex hormones should be determined. As our case, a high rate of testosterone, could guide us. Clinical examination reveals a short vagina, no uterus, and imaging techniques (ultrasound, CT, MRI) confirm the absence of the uterus and ovaries. Like in our case, intra- abdominal undescended testes, could be revealed by pelvic MRI [5]. Afterwards the karyotype has to be mapped (46XY) in order to differentiate the Androgen Insensitivity Syndrome from other genetic abnormalities; such as Klinefelter syndrome (46XXY), Turner syndrome (45XO), mixed gonadal dyssynergia (45XO/46XY) or tetragametic chimerism (46XX/46XY). The mutated androgen receptor gene may also be found [6]. Then, the difficulty of assigning sex turns out to be a real challenge, especially when it is a syndrome of insensitivity to androgens incomplete, where there is a wild variety of sexual ambiguity. While Complete Androgen Insensitivity Syndrome is rare. The vast majority of them patients kept seeing themselves as women as was the case with our patient [7]. Furthermore the orchiectomy should be done to avoid risk of malignancy of undescended intra-abdominal testes (3.6% at 25 years old, and 33% at 50 years old, according to various studies). In our case the laparoscopic approach was considered the best option, being a minimally invasive procedure with low associated morbidity. Thus, since the testes provide the natural levels of estrogens through the aromatizing of testosterone, hormonal substitution therapy can be administered, reason why our patient were referred to endocrinology department. In addition, the last issue is whether or not to disclose the entire pathology to the patient. In our case, the patient was not made aware of the disease, in order to spare more confusion and pain, as the self-image was clearly of a heterosexual woman. Therefore, our patient chose to not share her illness with her family. Whilst a great majority of Moroccan society still considers that everything « sex-related » is a taboo, therefore the family had no role as psychological supports. It was strongly recommended in order to help our patient to overcome the impact [8].

## Conclusion

Close collaboration between gynecologist, endocrinologist and psychologist is essential for proper management of complete insensitivity syndrome androgen. Due to the risk of degeneration of the gonad, castration be performed. An estrogen - progestin replacement therapy will be introduced to prevent the regression of secondary sexual characteristics and consequences of estrogen deficiency, also to preserve normal sexual activity. Finally, psychological support, with a collaboration between doctors, psychologists and family, remains indispensable.
